# ANTIHYPERTENSIVE DEPRESCRIBING AND COGNITIVE FUNCTION IN VA NURSING HOME RESIDENTS: A TARGET TRIAL EMULATION

**DOI:** 10.1093/geroni/igad104.2101

**Published:** 2023-12-21

**Authors:** Bocheng Jing, Xiaojuan Liu, Michelle Odden, Chintan Dave, Laura Graham, Yongmei Li, Kathy Fung, Michael Steinman

**Affiliations:** NCIRE, San Francisco VA Medical Center, UCSF, San Francisco, California, United States; Stanford University, Stanford, California, United States; Stanford University, Stanford, California, United States; Rutgers University, New Brunswick, New Jersey, United States; VA Palo Alto Health Care System, Palo Alto, California, United States; Stanford University, Stanford, California, United States; San Francisco VA Medical Center, San Francisco, California, United States; University of California, San Francisco, San Francisco, California, United States

## Abstract

Deprescribing is the practice of discontinuing or reducing medication that no longer provides benefits or causes harm. While deprescribing antihypertensives for frail elderly patients has gained interest, its impact on patient-centered outcomes such as cognitive function remains unknown. We emulated a target trial involving 45,183 Veteran Affairs nursing home residents aged ≥65 with stays ≥12 weeks between 2006-2019. Eligible residents must have a 4-week stable medication use period; they were allocated to the deprescribing/control arms, and censored at death, discharge, or two-year follow-up for intention-to-treat analysis. We used the Cognitive Function Scale (CFS) to assess cognitive outcomes, categorizing residents into cognitively intact, mild-, moderate-, and severely-impaired groups. We used an ordinal generalized linear-mixed model to analyze the effect of deprescribing on CFS, adjusting for confounders with 47 baseline covariates using inverse probability of the treatment weights. 1,290 and 11,354 patients were in deprescribing/control arms. At baseline, deprescribing was associated with an increased risk of worse cognitive function (OR=1.168 for being in a higher CFS category [95% CI:1.115-1.224]). Overall, cognition worsened in both arms over time (OR=1.007 per week for progression to higher CFS category, 95% CI:1.006-1.008). However, deprescribing mitigated the time trend, and the residents in the deprescribing arm had significantly decreased odds (improved CFS) at 0.996/week (95% CI: 0.995-0.997), 0.900 (95%CI: 0.881-0.919) over 26-weeks, and 0.811 (95% CI: 0.777-0.845) over 52-weeks. Our findings suggest that deprescribing is associated with a slower decline in cognitive function and can help inform future deprescribing interventions for BP management in nursing home residents.

